# Platelet-Rich Fibrin Increases CXCL8 Expression in Gingival Fibroblasts

**DOI:** 10.3390/biomedicines12061326

**Published:** 2024-06-14

**Authors:** Atefe Imani, Layla Panahipour, Natalia dos Santos Sanches, Lei Wang, Reinhard Gruber

**Affiliations:** 1 Department of Oral Biology, University Clinic of Dentistry, Medical University of Vienna, 1090 Vienna, Austria; dr_a_imani@hotmail.com (A.I.); layla.panahipour@meduniwien.ac.at (L.P.); natalia.s.sanches@unesp.br (N.d.S.S.); wanglei20111213@163.com (L.W.); 2 Department of Diagnosis and Surgery, Araçatuba Dental School of Sao Paulo, Sao Paulo 16015-050, Brazil; 3 Wenzhou Institute, University of Chinese Academy of Sciences, Wenzhou 325000, China; 4 Department of Periodontology, School of Dental Medicine, University of Bern, 3010 Bern, Switzerland; 5 Austrian Cluster for Tissue Regeneration, 1200 Vienna, Austria

**Keywords:** platelet-rich fibrin, gingival fibroblast, CXCL8, inflammation, bioassay

## Abstract

Platelet-rich fibrin (PRF), the coagulated plasma of fractionated blood, is widely used to support tissue regeneration in dentistry, and the underlying cellular and molecular mechanisms are increasingly being understood. Periodontal connective tissues steadily express CXCL8, a chemokine that attracts granulocytes and lymphocytes, supporting homeostatic immunity. Even though PRF is considered to dampen inflammation, it should not be ruled out that PRF increases the expression of CXCL8 in gingival fibroblasts. To test this hypothesis, we conducted a bioassay where gingival fibroblasts were exposed to PRF lysates and the respective serum. We show here that PRF lysates and, to a lesser extent, PRF serum increased the expression of CXCL8 by the gingival fibroblasts, as confirmed by immunoassay. SB203580, the inhibitor of p38 mitogen-activated protein kinase, reduced CXCL8 expression. Consistently, PRF lysates and, to a weaker range, the PRF serum also caused phosphorylation of p38 in gingival fibroblasts. Assuming that PRF is a rich source of growth factors, the TGF-β receptor type I kinase inhibitor SB431542 decreased the PRF-induced expression and translation of CXCL8. The findings suggest that PRF lysates and the respective serum drive CXCL8 expression by activating TGF-β and p38 signaling in gingival fibroblasts.

## 1. Introduction

Platelet-rich fibrin is produced from coagulated blood upon centrifugation [1]. A characteristic of PRF is the yellow plasma rich in platelets and leucocytes that, depending on the protocol, accumulate and are entrapped in the fibrin-rich matrix [1]. The coagulated yellow plasma is separated from the red clot and squeezed between two plates, resulting in a solid PRF membrane and a liquid PRF serum. Clinically, it is usually the solid PRF membrane that is applied to support regeneration, for instance, in ridge preservation after tooth extraction [2] and to treat periodontal intrabony defects or gingival recessions [3,4]. The PRF serum, often termed “exudate”, is another growth factor source not entrapped in the fibrin-rich matrix of the PRF membrane [5,6]. PRF is effective for two main reasons: firstly, PRF serves as a reservoir of growth factors and other bioactive molecules, and secondly, PRF membrane provides a three-dimensional scaffold that supports the ingrowth of the cells supposed to be involved in the repair process. Neutrophils are attracted to penetrate the fibrin-rich matrix, where they exert their immunological function. This process is central to wound healing and bone regeneration.

CXCL8, initially considered to be a cytokine, namely interleukin-8, is a significant driver of neutrophil recruitment to inflammatory sites, including damaged or infected areas [7]. CXCL8 further supports granulocyte phagocytosis, a process mediated by the G protein-coupled serpentine receptors CXCR1 and CXCR2 [7]. CXCL8 expression is a double-edged sword: (i) it supports local immunity being constantly expressed at a low level, (ii) or at transient higher levels during wound healing, (iii) however, when chronically expressed at high levels, CXCL8 becomes a key mediator associated with pathological inflammation and overaccentuated neutrophil recruitment and degranulation, overall causing tissue damage [7]. It is, therefore, not surprising that in healthy periodontal tissues, CXCL8 is regularly expressed to control tissue homeostasis, but CXCL8 is increasingly expressed in the pathological conditions of periodontitis [8,9,10] and periimplantitis [11]. Importantly, evidence suggests that fibroblasts are the primary source of CXCL8 in periodontitis [10] and periimplantitis [11]. Thus, CXCL8 became a significant target of in vitro research in an overall attempt to understand its regulation in gingival fibroblasts.

The question arises if PRF can modulate CXCL8 expression in gingival fibroblasts. Support for this assumption comes from observations that platelet-rich plasma prepared from anticoagulated blood enhanced the expression of CXCL8 in tenocytic [12] and synovial fibroblasts [13]. Moreover, PRF could stimulate CXCL8 expression in gingival keratinocytes [14]. Thus, existing research with platelet-rich plasma led us to assume that PRF can also drive the expression of CXCL8 in gingival fibroblasts. Moreover, PRF is a rich source of TGF-β1 [6,15,16], and TGF-β1 can induce CXCL8 production in renal cells [17], prostate cancer cells [18,19], and bronchial epithelial cells [19,20], breast cancer cells [19] and osteosarcoma cell lines [19]. However, similar research on how PRF changes CXCL8 expression in gingival fibroblasts has not been performed so far. Moreover, it remains open if the changes in CXCL8 expression observed with tenocytic [12] and synovial fibroblasts [13] were caused by the TGF-β activity of the platelet-rich plasma. Another open question is if the expression is mediated via the MAPK signaling pathway, particularly p38, as this pathway was involved in CXCL8 expression caused by viruses in myeloid cells [21], and by cytokine-exposed intestinal epithelial cells [22] and lung fibroblasts [23]. Moreover, CXCL8 expression is a downstream event of the nuclear factor-kappa B (NF-kappa B) family of transcription factor signaling [24]. It is thus relevant to understand the underlying molecular mechanisms of how PRF and the serum fraction drive CXCL8 expression in gingival fibroblasts.

Therefore, the aim of the present research was to study the impact of PRF lysates and PRF serum on CXCL8 expression in gingival fibroblasts and to analyze the possible involvement of TGF-β receptor type I kinase signaling. This aim was driven by the concept that PRF may exert its beneficial effects by modulating the local immunity and, thus, the defense of the periodontal tissues undergoing regeneration. We have consequently tested the hypothesis that PRF lysates and PRF serum increase CXCL8 expression in gingival fibroblasts.

## 2. Material and Methods

### 2.1. Gingival Fibroblasts

The Ethics Committee of the Medical University of Vienna (#631/2007) approved the isolation of fibroblasts from gingiva explants obtained during third molar surgery. The patients were told about the proposed research and gave their consent. We isolated cells from three explants and used the gingival fibroblasts at low passage. Gingival fibroblasts were expanded in αMEM (Sigma–Aldrich, St. Louis, MO, USA) supplemented with 10% fetal calf serum (Bio&Sell GmbH, Nuremberg, Germany) and 1% antibiotics (Sigma). The gingival fibroblasts were plated at 30,000 cells/cm^2^ one day before exposure.

### 2.2. PRF Lysates and PRF Serum

The preparation of PRF was approved by the Ethical Committee of the Medical University of Vienna (#1644/2018). Glass tubes (Bio-PRF, Venice, FL, USA) supporting blood coagulation were subjected to 700 g for 8 min (Z306 Hermle, Universal Centrifuge, Wehingen, Germany). The yellow PRF clot was removed and squeezed between two metal plates. The PRF serum fraction was collected and stored frozen. One cm of the PRF membrane was used per mL of serum-free medium to prepare PRF lysates. PRF membranes underwent two freeze-thawing cycles at −80 °C and RT, followed by 30 s of sonication (Sonopuls 2000.2, Bandelin electronic, Berlin, Germany). After centrifugation at 15,000× *g* for 10 min, aliquots of the PRF lysates were frozen for less than one month. PRF lysates were exposed to 72 °C and 95 °C for 10 min for indicated experiments.

### 2.3. Treatments of Cells

Gingival fibroblasts were treated with 30% PRF lysate and 10% PRF serum for 6 h in the incubator. Cells were also exposed to 10 ng/mL TGF-β1 (R&D Systems, Inc., Minneapolis, MN, USA) and 10 ng/mL IL1β and TNFα (ProSpec-Tany TechnoGene Ltd., Ness-Ziona, Israel). We also implemented 10 µM SB431542 (Calbiochem, Merck, Billerica, MA, USA), a TGF-β signaling inhibitor. To investigate the role of MAPK and PI3K/AKT signaling, gingival fibroblasts were incubated with the pharmacological inhibitors SB203580 (p38-inhibitor; Sigma), U0126 (ERK-inhibitor; Cell Signalling Technology, Beverly, MA, USA), SP600125 (JNK-inhibitor; Calbiochem, San Diego, CA, USA), and LY294002 (PI3K-inhibitor; Sigma), all at 10 μM. After 6 h treatment, gene expression was analyzed.

### 2.4. Reverse Transcription-Quantitative Real-Time PCR and Immunoassay

Following RNA extraction and DNAse digestion (EUR_x_, Gdańsk, Poland), the total RNA was reverse transcribed (LabQ, Labconsulting, Vienna, Austria), and cDNA was amplified on a CFX Connect™ RealTime PCR Detection System (Bio-Rad Laboratories, Hercules, CA, USA). The transcription levels of target genes indicated in CXCL8 [25] (F-AACTTCTCCACAACCCTCTG, R-TTGGCAGCCTTCCTGATTTC) were normalized to GAPDH (F-AAGCCACATCGCTCAGACAC, R-GCCCAATACGACCAAATCC) using the ΔΔCt method. The human CXCL8 immunoassay was used as recommended (DY208; R&D Systems, Minneapolis, MN, USA).

### 2.5. Immunofluorescence Analysis

Gingival fibroblasts at 15,000 cells/cm^2^ seeded onto microscopic slides (Millicell^®^ EZ slides, Merck KGaA, Darmstadt, Germany) were serum-deprived overnight. The following day, the cells were exposed to 30% PRF lysates and 10% PRF serum for one hour. After paraformaldehyde fixation and blocking by 1% bovine serum albumin, cells were made permeable with 0.3% Triton X-100 (all Sigma–Aldrich, St. Louis, MO, USA). The first antibodies were raised against smad2/3 (D7G7 XP^®^) and NF-κB p65 (Cell Signaling Technology, Cambridge, UK). The secondary antibody was applied after overnight incubation of the first antibody at 4 °C (CS-4412, Cell Signaling Technology). Pictures were taken on a fluorescence microscope (Echo Revolve fluorescence microscope, San Diego, CA, USA).

### 2.6. Western Blot

Gingival fibroblasts were deprived of serum and later treated with 30% PRF lysate and 10% PRF serum for one hour. SDS cell extracts containing protease and phosphatase inhibitors (cOmplete ULTRA tablets and PhosSTOP; Roche, Mannheim, Germany) were separated by electrophoresis and proteins blotted onto PVDF membranes (Roche Diagnostics, Mannheim, Germany). After blocking, membranes were incubated with antibodies raised against phosphor-p38 and p38 (sc-166182 and sc-535; Santa Cruz Biotechnology, Inc., Dallas, TX, USA) before being exposed to the HRP detection antibody (CS-7074; Cell Signaling Technology, Cambridge, UK). Chemiluminescence signals (Clarity Western ECL Substrate) were visualized with a ChemiDoc imaging system (all Bio-Rad Laboratories, Inc., Hercules, CA, USA).

### 2.7. Statistical Analysis

All the experiments were performed at least three times. Different data points indicate independent experiments. Statistical analysis was a Ratio paired t-test for single comparisons performed using Prism v9 (GraphPad Software, La Jolla, CA, USA). The significance was set at *p* < 0.05.

## 3. Results

### 3.1. PRF Lysates and PRF Serum Increase CXCL8 in Gingival Fibroblasts

**Figure 1:**
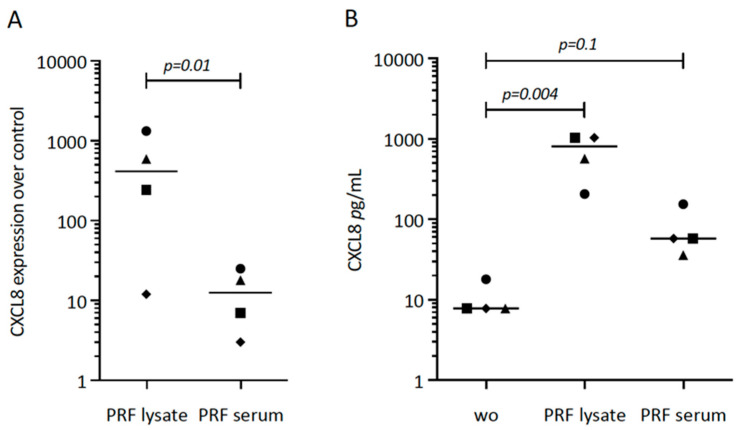
CXCL8 expression in gingival fibroblasts exposed to PRF lysate and PRF serum. (**A**) Gingival fibroblasts were exposed to PRF lysate and PRF serum for 6 h, and the expression of CXCL8 was normalized in the untreated cells. Data points represent four independent experiments. Statistical analysis was based on ratio paired t-tests, and *p*-values are indicated. (**B**) The respective CXCL8 levels in the conditioned medium were analyzed by immunoassay. To compare groups with wo (without, untreated cells), ratio paired t-tests were applied, and the *p*-values are shown.

First, we tried to understand the possible role of PRF lysates and the respective serum fraction on CXCL8 expression in gingival fibroblasts. Our original experiment showed a substantial increase of CXCL8 in cells exposed to PRF lysates (Figure 1A). Subsequently, we repeated the experiments with different donors, overall showing the enhanced expression of CXCL8 by gingival fibroblasts in response to PRF lysates and PRF serum (Figure 1A). The CXCL8 immunoassay confirmed the findings on the protein level (Figure 1B). It should be noticed, however, that PRF lysates and PRF serum are less potent than IL1β and TNFα to increase CXCL8 expression.

**Figure 2:**
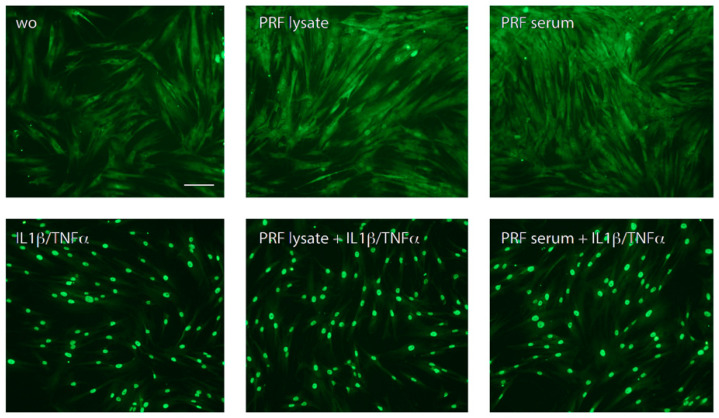
PRF lysate and PRF serum increased the nuclear translocation of p65 in gingival fibroblasts but less than when exposed to IL1β and TNFα. Gingival fibroblasts were left untreated (wo; without), exposed to PRF alone or in combination with IL1β and TNFα for 1 h. A faintly nuclear p65 labeling became apparent in cells exposed to PRF lysates and PRF serum; however, it requires IL1β and TNFα to provoke a complete accumulation of p65 in the nucleus. The scale bar represents 50 μm.

Consistent with the understanding that NFκB signaling pathways participate in CXCL8 expression [7,24], we studied p65 nuclear translocation, and indeed, PRF lysates and PRF serum-stimulated p65 nuclear translocation in gingival fibroblasts (Figure 2). These data suggest that PRF lysates and, to a large extent, the respective PRF serum also increase NFκB signaling and CXCL8 production in gingival fibroblasts.

### 3.2. PRF Lysate Requires TGF-β, p38 and PI3K Signaling to Drive CXCL8 Expression

**Figure 3:**
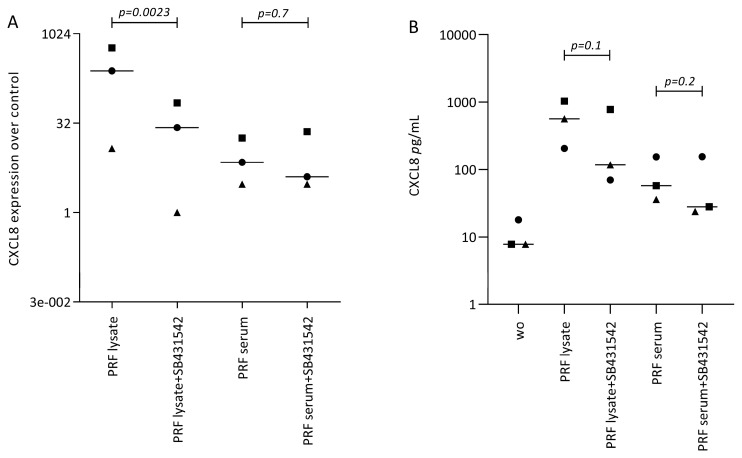
SB431542 reduces the PRF lysate-induced CXCL8 expression in gingival fibroblasts. (**A**) Gingival fibroblasts were exposed to PRF lysate and PRF serum with and without the TGF-β receptor type I kinase-inhibitor SB431542 for 6 h and the expression of CXCL8 was normalized to the untreated cells. Data points represent three independent experiments. Statistical analysis was based on ratio paired t-tests, and *p*-values are indicated. (**B**) The corresponding CXCL8 concentration in the conditioned medium was analyzed by immunoassay. To compare groups with wo (without or untreated cells), ratio paired t-tests were applied, and the *p*-values are indicated.

**Figure 4:**
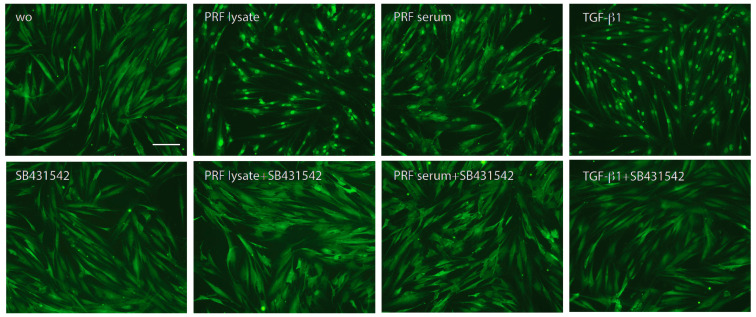
Nuclear translocation of Smad2/3 in response to the PRF lysate and PRF serum. Gingival fibroblasts were exposed to PRF lysate and PRF serum with and without the TGF-β receptor type I kinase-inhibitor SB431542. Recombinant TGF-β1 served as a positive control. Without (wo) is the serum-free medium alone. The positive signals induced by PRF lysate and PRF serum are blocked by SB431542 overall. The scale bar represents 50 μm.

Following, we treated gingival fibroblasts with PRF lysate and serum in the presence of SB431542 to block TGF-β signaling. SB431542 decreased the PRF lysate-induced CXCL8 expression in gingival fibroblasts, suggesting that the effects of PRF lysates are partially mediated via the TGF-β receptor type I kinase-inhibitor (Figure 3A). Consistent with existing evidence [21,22,23], SB431542 lowered the CXCL8 production induced by PRF lysates on the protein level (Figure 3B). In assumption of the TGF-β activity intrinsic to PRF and its lysates, the nuclear translocation of Smad2/3 was examined. Immunofluorescence analysis confirmed that PRF lysates, and even though less pronounced, PRF serum could also induce the nuclear translocation Smad2/3 in gingival fibroblasts (Figure 4). In support of the underlying TGF-β receptor type activity, SB431542 blocked the Smad2/3 nuclear translocation (Figure 4).

**Figure 5:**
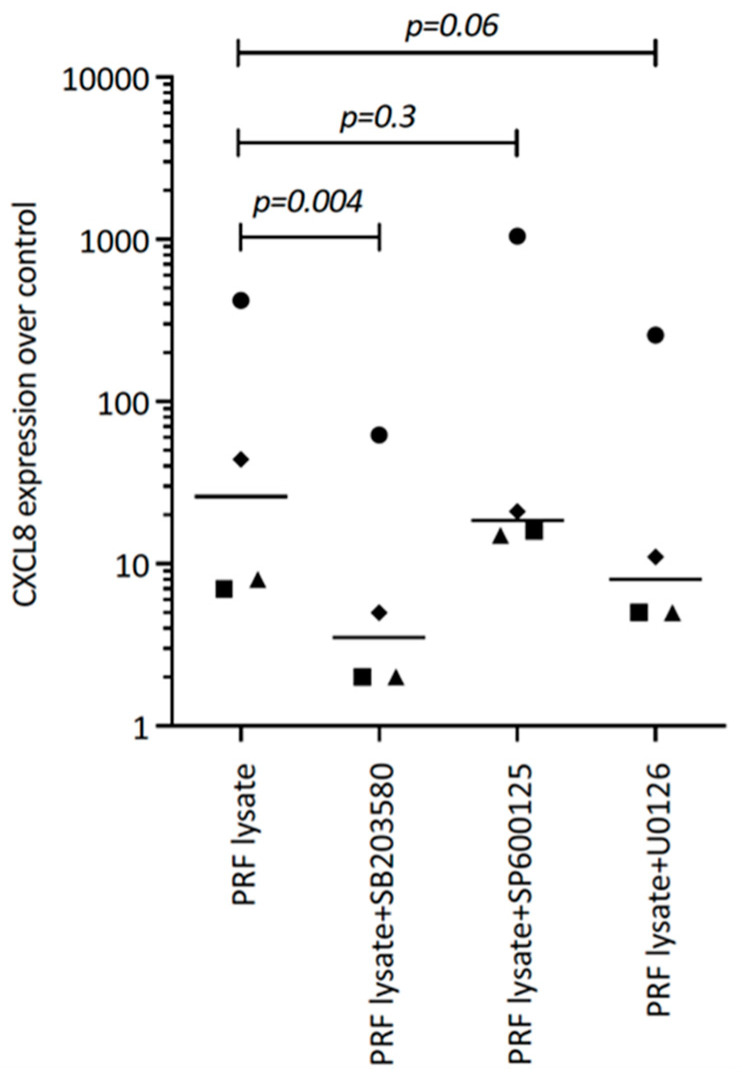
SB203580 reduces the PRF lysate-induced CXCL8 expression in gingival fibroblasts. Gingival fibroblasts were exposed to PRF lysate and PRF serum with and without the MAPK inhibitors (p38 signaling with SB203580, JNK with SP600125, and ERK with U0126) for 6 h, and the expression of CXCL8 was normalized to the untreated cells. Data points represent four independent experiments. Statistical analysis was based on ratio paired *t*-tests and *p*-values are indicated.

**Figure 6:**
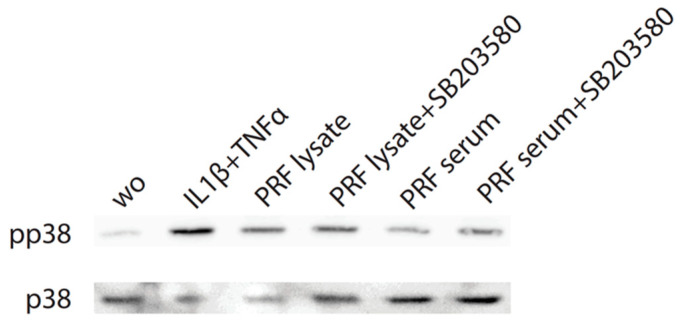
PRF lysates increase phosphorylation of p38 in gingival fibroblasts. Cells were treated with PRF lysates, PRF serum, and IL1β/TNFα and SB203580 before Western blot analysis was carried out for phospho-p38 and total p38. “wo” means without and represents unstimulated cells. SB203580 inhibits p38 catalytic activity but does not inhibit phosphorylation of p38 by upstream kinases.

Considering that TGF-β receptor signaling also involves non-Smad pathways [26], we studied other signaling cascades possibly mediating CXCL8 expression. We noticed that, in particular, blocking p38 signaling with SB203580 significantly reduced the forced CXCL8 expression, whereas blocking ERK with U0126 and JNK inhibition with SP600125 had no noticeable impact on CXCL8 expression (Figure 5). Support comes from our Western blot analysis showing that PRF lysates and also PRF serum increased the phosphorylation of p38 in gingival fibroblasts (Figure 6). These findings implicate that the p38 signaling pathway at least partially mediates the PRF-induced CXCL8 expression.

### 3.3. PRF Lysates Contain Heat-Sensitive Molecules Enhancing CXCL8 Expression

**Figure 7:**
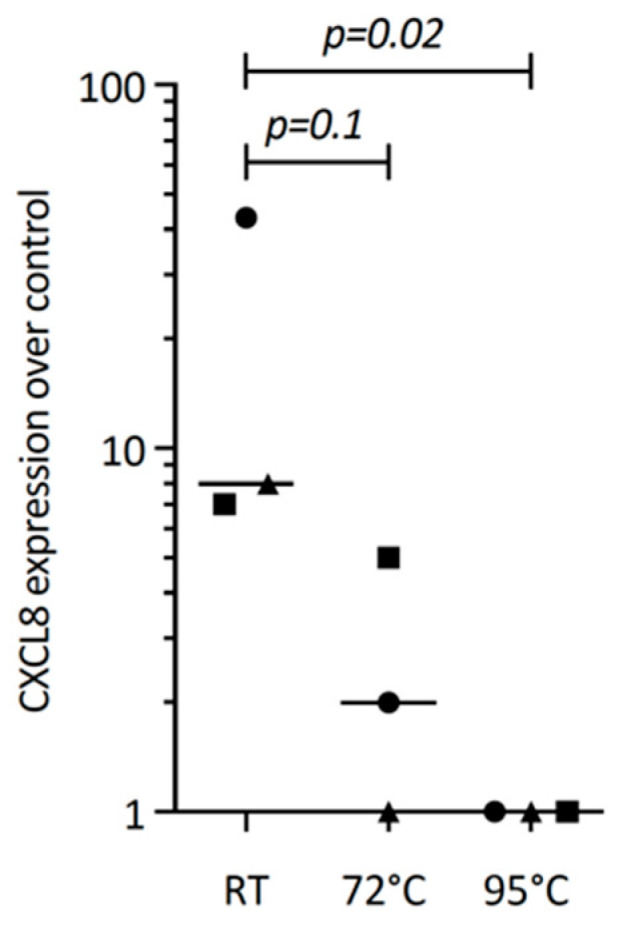
Heating reduces the PRF lysate-induced CXCL8 expression in gingival fibroblasts. Gingival fibroblasts were exposed to native PRF lysate, and PRF heated up to 72 °C and 95 °C for 10 min, and were incubated 6 h before measuring CXCL8 expression. Data points represent three independent experiments. Statistical analysis was based on ratio paired *t*-tests, and *p*-values are indicated.

The next question we asked was whether or not TGF-β1 within the PRF lysates drives CXCL8 expression. We could confirm that PRF lysates and TGF-β1 can both drive IL11 expression, a characteristic TGF-β1 target gene in gingival fibroblasts [15], similar to cardiac fibroblasts and other cells [27]. However, recombinant TGF-β1 failed to significantly push CXCL8 expression in gingival fibroblasts, suggesting that other PRF-derived molecules activating TGF-β receptor signaling are responsible for this observation. To understand the characteristics of these molecules, PRF lysates were heated. Heating PRF lysates at 72 °C and, in particular, at 95 °C for 10 min reduced the capacity of PRF to drive CXCL8 expression. These data suggest that PRF molecules responsible for CXCL8 expression are sensitive to high temperatures (Figure 7).

## 4. Discussion

PRF is basically a blood clot where the erythrocytes have been removed by centrifugation [1]. Even though clinical experience supports the use of PRF [2,3,4], explanations for the beneficial effects of PRF in the clinical setting remain at the level of a hypothesis. It is reasonable to suggest that PRF membranes serve as a natural scaffold. In this fibrin-rich matrix, platelets and leucocytes accumulate, serving as a reservoir for growth factors and other molecules potentially beneficial for wound healing and bone regeneration. It is also relevant to state that the PRF serum, often termed “exudate”, is another growth factor source being squeezed out of the PRF membrane [5,6]. In vitro bioassays have helped to translate how these growth factors affect the cell response, for instance, the proliferation and migration of fibroblasts as well as the stimulation of collagen synthesis [28,29]. These and other bioassays simulate the direct effect of PRF on the respective target cells. However, RNAseq revealed that PRF also affects fibroblast’s autocrine and paracrine functions [15]. In this context, we have identified PRF as causing a robust increase in IL11 [15] expression in gingival fibroblasts, an effect that can be traced back to the activation of TGF-β signaling provoked by PRF. IL11, in turn, is a potent pleiotropic cytokine that drives the gp130—IL6-family signaling pathway in various cell types with a prominent role in tissue homeostasis, including bone [30], also causing pathological changes related to fibrosis [31]. Our present goal is to identify further genes targeted by PRF that may be linked to the autocrine and paracrine activity of gingival fibroblasts, which is, in the present study, CXCL8.

CXCL8 is a lead chemokine that signals neutrophils where to move, thus driving the process of innate immunity, which is part of tissue defense and the early phase of regeneration [7]. CXCL8 is more than an inflammatory cytokine, as it is expressed in healthy periodontal tissues, suggesting its involvement in the permanent need for the periodontium to defend against the microbial burden of the oral cavity [10]. Moreover, once homeostasis shifts into the pathological stage, CXCL8 becomes a marker of periodontitis [8,9,10] and periimplantitis [11]. The fibroblasts are the primary producers of CXCL8 in periodontitis tissues [10]. In the present research, we observed that PRF lysates and, to a minor extent, the respective serum cause gingival fibroblasts to produce CXCL8 increasingly. Our data support research showing that CXCL8 in tenocytic [12], synovial fibroblasts [13] and gingival keratinocytes [14] is enhanced by platelet-rich plasma. However, when murine stromal cells are exposed to IL1β and TNFα, PRF lysates become potent inhibitors of cytokine production. Thus, opposite effects on human fibroblasts were observed in a mouse-based bioassay [32]. Interestingly, liquid PRF only caused a nonsignificant increase of IL1β and TNFα in human periodontal ligament fibroblasts [33]. The relevance of the present research, therefore, exceeds the primary finding of PRF-induced CXCL8 production in human gingival fibroblasts; our finding may lead to a rejection of our original hypothesis that PRF has anti-inflammatory activity, which is true in mouse models but not necessarily in human bioassays.

Considering that PRF contains a large spectrum of growth factors and other bioactive molecules, the question arises: Which factor is responsible for causing CXCL8 expression in gingival fibroblasts? We hypothesized TGF-β to be a potential candidate based on what we know about IL11 expression [15]. PRF is a rich source of TGF-β1 [6,15,16], and recombinant TGF-β1 was reported to induce CXCL8 production in renal cells [17], prostate cancer cells [18,19], bronchial epithelial cells [19,20], breast cancer cells [19], and osteosarcoma cell lines [19]. However, similar research on how PRF changes CXCL8 expression in gingival fibroblasts was not performed, and it remains unclear if the changes in CXCL8 expression observed with PRP and PRF in tenocytic [12], synovial fibroblasts [13], and gingival keratinocytes were caused by TGF-β activity [14]. Support for this hypothesis comes from our observation that blocking the TGF-β receptor type I kinase with SB431542 diminished the capacity of PRF lysates to drive CXCL8 expression. Moreover, and consistent with our previous studies related to IL11 expression [15], PRF lysates and, to a lesser extent, PRF serum initiated the nuclear translocation of the canonical smad2/3 transcription factor, again suggesting activation of TGF-β signaling. Nevertheless, we must reject our original hypothesis as recombinant TGF-β1 failed to drive CXCL8 expression in gingival fibroblast. We can, therefore, only assume that the molecular mechanism that drives PRF lysate-induced CXCL8 expression involves TGF-β receptor type I kinase signaling. However, it cannot simply be blamed on TGF-β activity. Future research is needed to identify the underlying molecular mechanisms driving the CXCL8 expression. One strategy is to perform an RNAseq to understand the overall change of the genetic signature of the gingival fibroblasts, including the expression of other chemokines and cytokines, as well as the regulation of the underlying signaling pathways—which provide insights into how PRF may exert its activity in the mesenchymal cell population.

The present study has limitations; for instance, we have noticed that PRF lysates consistently increase CXCL8 expression in gingival fibroblasts. However, PRF is significantly less effective than IL1β and TNFα in provoking CXCL8 expression. Thus, there remains speculation about whether the beneficial effects of PRF in a clinical scenario can be related to the enhanced CXCL8 expression in fibroblasts and the corresponding influx of neutrophils and other immune cells in vivo. The clinical relevance of our findings needs to be identified in future research. Moreover, and considering that we have not identified the molecules in PRF that cause the CXCL8 expression in the fibroblasts, we have to stay descriptive, sharing information that the biological activity of PRF is heat sensitive already at 72 °C. In this context, we were suspicious that TGF-β in PRF, an activity that drives IL11 expression, is also responsible for CXCL8 expression, an observation supported by SB431542 inhibition and Smad2/3 nuclear translocation.

Inhibition of p38 signaling with SB203580 almost completely blocks CXCL8 expression, a finding that is consistent with other work showing that SB203580 blocked the effect of *P. gingivalis* LPS on CXCL8 in periodontal ligament fibroblasts [34], in acid-induced esophageal epithelial cells [35] or *P. acnes* extracts-induced sebaceous gland cells [36]. These observations are overall consistent with what is reported for myeloid cells [21], epithelial cells [22], and lung fibroblasts [23]. However, since recombinant TGF-β1 failed to drive CXCL8 expression consistently but strongly increased the expected IL11 expression in our bioassay, we had to reject this hypothesis. In search of potential candidates, we studied CXCL12 being released from platelets and a potent heat-stable agonist of CXCL8 expression [37,38]. For instance, CXCL12 induces CXCL8 production in human cord blood-derived mast cells [39] and T-cell acute lymphoblastic leukemia cells [40]. Moreover, CXCL12-induced Smad3 nuclear translocation in human lung fibroblasts [41]. However, in our bioassay, recombinant CXCL12 failed to change CXCL8 expression. Therefore, we must continue our search for PRF-derived molecules responsible for enhancing CXCL8 expression by gingival fibroblasts.

In summary, the present research adds another piece of information on PRF research, showing that PRF membrane lysates and, to a lesser extent, the PRF serum fraction can enhance the CXCL8 production of gingival fibroblasts. This process requires TGF-β signaling but is not directly mediated by growth factors.

## Data Availability

The original contributions presented in the study are included in the article. Further inquiries can be directed to the corresponding author.

## References

[1] Miron R.J., Fujioka-Kobayashi M., Sculean A., Zhang Y. (2023). Optimization of platelet-rich fibrin. Periodontol. 2000.

[2] Castro A.B., Van Dessel J., Temmerman A., Jacobs R., Quirynen M. (2021). Effect of different platelet-rich fibrin matrices for ridge preservation in multiple tooth extractions: A split-mouth randomized controlled clinical trial. J. Clin. Periodontol..

[3] Silva F., Chauca-Bajana L., Caponio V.C.A., Cueva K.A.S., Velasquez-Ron B., Padin-Iruegas M.E., Almeida L.L., Lorenzo-Pouso A.I., Suarez-Penaranda J.M., Perez-Sayans M. (2024). Regeneration of periodontal intrabony defects using platelet-rich fibrin (PRF): A systematic review and network meta-analysis. Odontology.

[4] Tavelli L., Chen C.J., Barootchi S., Kim D.M. (2022). Efficacy of biologics for the treatment of periodontal infrabony defects: An American Academy of Periodontology best evidence systematic review and network meta-analysis. J. Periodontol..

[5] Li X., Yang H., Zhang Z., Yan Z., Lv H., Zhang Y., Wu B. (2018). Platelet-rich fibrin exudate promotes the proliferation and osteogenic differentiation of human periodontal ligament cells in vitro. Mol. Med. Rep..

[6] Castro A.B., Cortellini S., Temmerman A., Li X., Pinto N., Teughels W., Quirynen M. (2019). Characterization of the Leukocyte- and Platelet-Rich Fibrin Block: Release of Growth Factors, Cellular Content, and Structure. Int. J. Oral. Maxillofac. Implant..

[7] Cambier S., Gouwy M., Proost P. (2023). The chemokines CXCL8 and CXCL12: Molecular and functional properties, role in disease and efforts towards pharmacological intervention. Cell. Mol. Immunol..

[8] Frasheri I., Heym R., Ern C., Summer B., Hennessen T.G., Hogg C., Reichl F.X., Folwaczny M. (2022). Salivary and gingival CXCL8 correlation with periodontal status, periodontal pathogens, and smoking. Oral Dis..

[9] Finoti L.S., Nepomuceno R., Pigossi S.C., Corbi S.C., Secolin R., Scarel-Caminaga R.M. (2017). Association between interleukin-8 levels and chronic periodontal disease: A PRISMA-compliant systematic review and meta-analysis. Medicine.

[10] Williams D.W., Greenwell-Wild T., Brenchley L., Dutzan N., Overmiller A., Sawaya A.P., Webb S., Martin D., Genomics N.N., Computational Biology C. (2021). Human oral mucosa cell atlas reveals a stromal-neutrophil axis regulating tissue immunity. Cell.

[11] Mo J.J., Lai Y.R., Huang Q.R., Li Y.R., Zhang Y.J., Chen R.Y., Qian S.J. (2024). Single-cell sequencing identifies inflammation-promoting fibroblast-neutrophil interaction in peri-implantitis. J. Clin. Periodontol..

[12] Andia I., Rubio-Azpeitia E. (2014). Angiogenic and innate immune responses triggered by PRP in tendon cells are not modified by hyperuricemia. Muscles Ligaments Tendons J..

[13] Assirelli E., Filardo G., Mariani E., Kon E., Roffi A., Vaccaro F., Marcacci M., Facchini A., Pulsatelli L. (2015). Effect of two different preparations of platelet-rich plasma on synoviocytes. Knee Surg. Sports Traumatol. Arthrosc..

[14] Kasnak G., Fteita D., Jaatinen O., Kononen E., Tunali M., Gursoy M., Gursoy U.K. (2019). Regulatory effects of PRF and titanium surfaces on cellular adhesion, spread, and cytokine expressions of gingival keratinocytes. Histochem. Cell Biol..

[15] Di Summa F., Kargarpour Z., Nasirzade J., Stahli A., Mitulovic G., Panic-Jankovic T., Koller V., Kaltenbach C., Muller H., Panahipour L. (2020). TGFbeta activity released from platelet-rich fibrin adsorbs to titanium surface and collagen membranes. Sci. Rep..

[16] Fujioka-Kobayashi M., Miron R.J., Hernandez M., Kandalam U., Zhang Y., Choukroun J. (2017). Optimized Platelet-Rich Fibrin With the Low-Speed Concept: Growth Factor Release, Biocompatibility, and Cellular Response. J. Periodontol..

[17] Qi W., Chen X., Polhill T.S., Sumual S., Twigg S., Gilbert R.E., Pollock C.A. (2006). TGF-beta1 induces IL-8 and MCP-1 through a connective tissue growth factor-independent pathway. Am. J. Physiol. Renal Physiol..

[18] Lu S., Dong Z. (2006). Characterization of TGF-beta-regulated interleukin-8 expression in human prostate cancer cells. Prostate.

[19] Fong Y.C., Maa M.C., Tsai F.J., Chen W.C., Lin J.G., Jeng L.B., Yang R.S., Fu W.M., Tang C.H. (2008). Osteoblast-derived TGF-beta1 stimulates IL-8 release through AP-1 and NF-kappaB in human cancer cells. J. Bone Miner. Res..

[20] Krick S., Baumlin N., Aller S.P., Aguiar C., Grabner A., Sailland J., Mendes E., Schmid A., Qi L., David N.V. (2017). Klotho Inhibits Interleukin-8 Secretion from Cystic Fibrosis Airway Epithelia. Sci. Rep..

[21] Fu Y., Yip A., Seah P.G., Blasco F., Shi P.Y., Herve M. (2014). Modulation of inflammation and pathology during dengue virus infection by p38 MAPK inhibitor SB203580. Antiviral Res..

[22] Sunil Y., Ramadori G., Raddatzc D. (2010). Influence of NFkappaB inhibitors on IL-1beta-induced chemokine CXCL8 and -10 expression levels in intestinal epithelial cell lines: Glucocorticoid ineffectiveness and paradoxical effect of PDTC. Int. J. Color. Dis..

[23] Higham A., Singh D. (2021). Dexamethasone and p38 MAPK inhibition of cytokine production from human lung fibroblasts. Fundam. Clin. Pharmacol..

[24] Richmond A. (2002). Nf-kappa B, chemokine gene transcription and tumour growth. Nat. Rev. Immunol..

[25] Mao P., Wu S., Li J., Fu W., He W., Liu X., Slutsky A.S., Zhang H., Li Y. (2015). Human alveolar epithelial type II cells in primary culture. Physiol. Rep..

[26] Zhang Y.E. (2009). Non-Smad pathways in TGF-beta signaling. Cell Res..

[27] Schafer S., Viswanathan S., Widjaja A.A., Lim W.W., Moreno-Moral A., DeLaughter D.M., Ng B., Patone G., Chow K., Khin E. (2017). IL-11 is a crucial determinant of cardiovascular fibrosis. Nature.

[28] Sterczala B., Chwilkowska A., Szwedowicz U., Kobielarz M., Chwilkowski B., Dominiak M. (2022). Impact of APRF+ in Combination with Autogenous Fibroblasts on Release Growth Factors, Collagen, and Proliferation and Migration of Gingival Fibroblasts: An In Vitro Study. Materials.

[29] Bucur M., Constantin C., Neagu M., Zurac S., Dinca O., Vladan C., Cioplea M., Popp C., Nichita L., Ionescu E. (2019). Alveolar blood clots and platelet-rich fibrin induce in vitro fibroblast proliferation and migration. Exp. Ther. Med..

[30] Dong B., Zhu J., Chen X., Jiang H., Deng Y., Xu L., Wang Y., Li S. (2023). The Emerging Role of Interleukin-(IL)-11/IL-11R in Bone Metabolism and Homeostasis: From Cytokine to Osteokine. Aging Dis..

[31] Cook S.A. (2023). Understanding interleukin 11 as a disease gene and therapeutic target. Biochem. J..

[32] Kargarpour Z., Nasirzade J., Panahipour L., Miron R.J., Gruber R. (2021). Platelet-Rich Fibrin Decreases the Inflammatory Response of Mesenchymal Cells. Int. J. Mol. Sci..

[33] Zheng S., Zhang X., Zhao Q., Chai J., Zhang Y. (2020). Liquid platelet-rich fibrin promotes the regenerative potential of human periodontal ligament cells. Oral Dis..

[34] Zhang Y., Li X. (2015). Lipopolysaccharide-regulated production of bone sialoprotein and interleukin-8 in human periodontal ligament fibroblasts: The role of toll-like receptors 2 and 4 and the MAPK pathway. J. Periodontal Res..

[35] Rafiee P., Nelson V.M., Manley S., Wellner M., Floer M., Binion D.G., Shaker R. (2009). Effect of curcumin on acidic pH-induced expression of IL-6 and IL-8 in human esophageal epithelial cells (HET-1A): Role of PKC, MAPKs, and NF-kappaB. Am. J. Physiol. Gastrointest. Liver Physiol..

[36] Huang Y.C., Yang C.H., Li T.T., Zouboulis C.C., Hsu H.C. (2015). Cell-free extracts of Propionibacterium acnes stimulate cytokine production through activation of p38 MAPK and Toll-like receptor in SZ95 sebocytes. Life Sci..

[37] Chatterjee M., Gawaz M. (2013). Platelet-derived CXCL12 (SDF-1alpha): Basic mechanisms and clinical implications. J. Thromb. Haemost..

[38] Takekoshi T., Ziarek J.J., Volkman B.F., Hwang S.T. (2012). A locked, dimeric CXCL12 variant effectively inhibits pulmonary metastasis of CXCR4-expressing melanoma cells due to enhanced serum stability. Mol. Cancer Ther..

[39] Lin T.J., Issekutz T.B., Marshall J.S. (2001). SDF-1 induces IL-8 production and transendothelial migration of human cord blood-derived mast cells. Int. Arch. Allergy Immunol..

[40] Scupoli M.T., Donadelli M., Cioffi F., Rossi M., Perbellini O., Malpeli G., Corbioli S., Vinante F., Krampera M., Palmieri M. (2008). Bone marrow stromal cells and the upregulation of interleukin-8 production in human T-cell acute lymphoblastic leukemia through the CXCL12/CXCR4 axis and the NF-kappaB and JNK/AP-1 pathways. Haematologica.

[41] Lin C.H., Shih C.H., Lin Y.C., Yang Y.L., Chen B.C. (2018). MEKK1, JNK, and SMAD3 mediate CXCL12-stimulated connective tissue growth factor expression in human lung fibroblasts. J. Biomed. Sci..

